# Preferences of Chinese Dermatologists for Large Language Model Responses in Clinical Psoriasis Scenarios: A Nationwide Cross‐Sectional Survey in China

**DOI:** 10.1002/hcs2.70057

**Published:** 2026-02-11

**Authors:** Jungang Yang, Jingkai Xu, Xuejiao Song, Chengxu Li, Lili Chen, Lingbo Bi, Tingting Jiang, Xianbo Zuo, Yong Cui

**Affiliations:** ^1^ China‐Japan Friendship School of Clinical Medicine Peking University Beijing China; ^2^ Department of Dermatology China‐Japan Friendship Hospital Beijing China; ^3^ Graduate School, Chinese Academy of Medical Sciences and Peking Union Medical College Beijing China; ^4^ School of Life Science and Technology Southeast University Nanjing China

**Keywords:** dermatology, large language model, model evaluation

## Abstract

**Background:**

Large language models (LLMs) have shown considerable promise in supporting clinical decision‐making. However, their adoption and evaluation in dermatology remains limited. This study aimed to explore the preferences of Chinese dermatologists regarding LLM‐generated responses in clinical psoriasis scenarios and to assess how they prioritize key quality dimensions, including accuracy, traceability, and logicality.

**Methods:**

A cross‐sectional, web‐based survey was conducted between December 25, 2024, and January 22, 2025, following the Checklist for Reporting Results of Internet E‐Surveys guidelines. A total of 1247 valid responses were collected from practicing dermatologists across 33 of China's provincial‐level administrative divisions. Participants evaluated responses to five categories of clinical questions (etiology, clinical presentation, differential diagnosis, treatment, and case study) generated by five LLMs: ChatGPT‐4o, Kimi.ai, Doubao, ZuoYiGPT, and Lingyi‐agent. Statistical associations between participant characteristics and model preferences were examined using chi‐square tests.

**Results:**

ChatGPT‐4o (Model 1) emerged as the most preferred model across all clinical tasks, consistently receiving the highest number of votes in case study (*n* = 740), clinical presentation (*n* = 666), differential diagnosis (*n* = 707), etiology (*n* = 602), and treatment (*n* = 656). Significant variation in model preference by professional title was observed only for the differential diagnosis task (*χ* ^2^ = 21.13, *df* = 12, *p* = 0.0485), while no significant differences were found across hospital tiers (*p* > 0.05). In terms of evaluation dimensions, accuracy was most frequently rated as “very important” (*n* = 635). A significant association existed between hospital tier and the most valued dimension (*χ* ^2^ = 27.667, *df* = 9, *p* = 0.0011), with dermatologists in primary hospitals prioritizing traceability more than their peers in higher‐tier hospitals. No significant associations were found across professional titles (*p* = 0.127).

**Conclusions:**

Chinese dermatologists suggest a strong preference for ChatGPT‐4o over domestic LLMs in psoriasis‐related clinical tasks. While accuracy remains the primary criterion, traceability and logicality are also critical, particularly for clinicians in lower‐tier hospitals. These findings suggest that future clinical LLMs should prioritize not only content accuracy but also source transparency and structural clarity to meet the diverse needs of different clinical settings.

## Graphical abstract

In a nationwide survey of 1247 Chinese dermatologists, ChatGPT‐4o was the most preferred large language model (LLM) for psoriasis‐related clinical tasks. Accuracy emerged as the top evaluation criterion for LLM‐generated responses. All icons were made by Freepik and Maan Icons from https://www.flaticon.com/

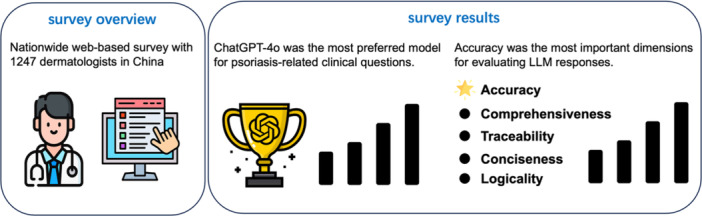

**Abbreviations**
AI: artificial intelligence
LLM: large language model
SD: standard deviation

## 1 Introduction

The rapid development of artificial intelligence (AI) is progressively transforming clinical practice across various medical fields [1]. Large language models (LLMs), which are trained on extensive textual datasets, have shown considerable promise in supporting disease diagnosis, treatment planning, and medical knowledge synthesis. Medical‐specific LLMs are particularly anticipated to enhance both the efficiency and accuracy of clinical decision‐making [2, 3, 4, 5]. Despite a growing body of research exploring medical students' and healthcare workers' knowledge, attitudes, and practices regarding LLMs [6, 7], the real‐world adoption of these models remains limited. This is, in part, due to variability in clinicians' perceptions, acceptance, and preferences for usage, which present significant barriers to widespread implementation.

Dermatology is a specialty that relies heavily on clinical expertise and visual judgment. It faces persistent challenges, including a broad range of conditions, overlapping symptomatology, and high misdiagnosis rates, particularly in primary hospitals [8]. Consequently, decision‐support systems are urgently needed to enhance diagnostic accuracy and streamline clinical workflows. Although LLMs offer promising solutions, there remains a paucity of research examining how dermatologists, as end users, perceive and evaluate these tools. Specifically, few studies have examined how clinicians assess LLM‐generated responses in clinical scenarios and how they prioritize key quality dimensions, such as accuracy, traceability, and logical coherence.

A growing number of LLMs have been integrated into medical applications in China, including both general‐purpose models (e.g., OpenAI's ChatGPT and Kimi.ai) and domain‐specific models (e.g., ZuoYiGPT and Baidu's Lingyi‐agent). While existing studies have examined the performance of LLMs in terms of accuracy, generalizability, and interpretability [9, 10, 11], a critical gap remains in systematic comparisons of different models within dermatology. Notably, there is a lack of user‐centered evaluations that involve real clinical tasks, as well as insufficient data on how dermatologists prioritize various response quality dimensions. These gaps underscore the need for further investigation.

Therefore, we conducted a nationwide survey of Chinese dermatologists to examine their preferences for LLM‐generated responses in clinical dermatology scenarios. Additionally, we explored how they prioritize key evaluation dimensions—such as accuracy, traceability, and logical coherence—when assessing content quality. This study aims to inform the development of clinically relevant LLMs and facilitate their effective integration into dermatological practice.

## 2 Methods

### 2.1 Study Design and Target Population

This cross‐sectional, web‐based survey was conducted in accordance with the Checklist for Reporting Results of Internet E‐Surveys guidelines to ensure quality and transparency in reporting [12]. The primary objective was to examine dermatologists' preferences and evaluations of responses generated by LLMs.

The target population consisted of actively practicing dermatologists from 33 of China's provincial‐level administrative divisions, representing a range of hospital tiers and professional titles. A convenience sampling approach was employed.

### 2.2 Questionnaire Development and Pilot Testing

The questionnaire was developed by a panel of three experts in dermatology and AI, who ensured the content validity, clinical relevance, and clarity of the items. Before formal distribution, a small‐scale pilot test was conducted to refine the questionnaire's logical flow and user interface. Upon completion of the survey, all LLM‐generated responses included in the questionnaire were independently evaluated by three dermatology and AI experts to assess key content dimensions, including accuracy, logicality, comprehensiveness, and traceability of sources. These expert assessments were then used to contextualize and interpret the user preference data in Section Conclusions, 4.

### 2.3 Survey Administration and Recruitment

The questionnaire was administered via the Questionnaire Star platform (https://www.wjx.cn/) and remained open for 4 weeks, from December 25, 2024, to January 22, 2025.

Participants were recruited through WeChat groups consisting of dermatologists affiliated with provincial and institutional quality‐control networks. The survey was openly accessible to members who met the inclusion criteria. Participation was voluntary, and no incentives, either monetary or nonmonetary, were provided.

### 2.4 Informed Consent and Data Protection

At the beginning of the survey, participants were provided with an information page detailing the study's purpose, estimated completion time, and assurances regarding anonymity and data confidentiality. No personally identifiable information was collected. All responses were anonymized and stored securely and were used exclusively for academic research purposes.

### 2.5 Questionnaire Structure and Quality Control

The questionnaire consisted exclusively of mandatory closed‐ended items, designed to capture participant demographics, model preferences across clinical scenarios, and evaluations of LLM response quality (Data S1). The clinical section included five categories of questions related to psoriasis: etiology, clinical presentation, differential diagnosis, treatment planning, and case analysis. These categories were chosen because they represent common clinical scenarios encountered in dermatologists' daily practice and are considered essential for effective psoriasis management.

The five LLMs included in this study (ChatGPT‐4o, Kimi.ai, Doubao, ZuoYiGPT, and Lingyi‐agent) are publicly available general‐purpose models. To the best of our knowledge, none of these models has been fine‐tuned using private medical corpora for this evaluation. Information regarding their training datasets and corpus coverage is limited to what has been publicly disclosed by their developers, with no additional technical details available to the authors. All survey questions were designed in Chinese, and participants were instructed to provide their responses in Chinese to maintain a consistent linguistic and clinical context for comparison across models.

To guide the evaluation of LLM‐generated responses, dermatologists were introduced to five key quality dimensions before completing the questionnaire. Participants were then asked to rate the importance of each dimension on a six‐point scale, ranging from “very unimportant” to “very important,” with only one dimension allowed to be rated as “very important.” Each dimension was explicitly defined to ensure a standardized understanding: (1) Accuracy: The correctness of the content and its freedom from factual errors; (2) Comprehensiveness: The extent to which the response fully addresses all relevant aspects of the question; (3) Conciseness: The succinctness of the response, avoiding unnecessary redundancy; (4) Logicality: The logical organization, coherence, and readability of the content; (5) Traceability: The inclusion of references or sources (e.g., websites, literature, or clinical guidelines) to support the response.

A unique WeChat ID verification mechanism was implemented to ensure one submission per participant, thereby preventing duplicate entries.

Participants were allowed to review and revise their answers before final submission. A completeness check was incorporated into the system to prompt users to complete all items before submission, followed by a summary screen for the final review of all responses.

### 2.6 Response Metrics and Inclusion Criteria

A total of 1325 responses were collected during the survey period, of which 1247 were deemed valid based on predefined criteria—specifically, that participants were actively practicing dermatologists. Since the Questionnaire Star platform does not provide data on survey page visits, the view rate and participation rate could not be determined. Only unique, valid entries were included in the final analysis.

### 2.7 Ethical Considerations

Given that this study involved an anonymous, voluntary survey of healthcare professionals and did not collect patient data or personally identifiable information, formal ethical approval was not required. The study adhered to the principles outlined in the Declaration of Helsinki, including informed consent and the protection of participant confidentiality.

### 2.8 Statistical Analysis

Continuous variables were summarized as means and standard deviations (SD), while categorical variables were presented as frequencies and percentages. Chi‐square tests of independence were used to assess associations between physician characteristics (professional title and hospital tier) and their preferences for models and evaluation priorities. A *p*‐value of less than 0.05 was considered statistically significant. All statistical analyses were performed using R (version 4.3.1), and visualizations were created using the ggplot2 package for bar charts and the fmsb package for radar charts.

## 3 Results

### 3.1 Basic Characteristics of the Participating Dermatologists

A total of 1247 dermatologists participated in the study. Their demographic and professional characteristics are summarized in Table 1.

**Table 1:** Demographic and professional characteristics of participants.

Characteristics	Participants
No. of unique individuals	1247
Age, y (mean ± SD)	43.96 ± 16.20
Sex (*n*, %)	
Male	496 (39.78%)
Female	751 (60.22%)
Hospital tiers (*n*, %)	
Class A tertiary hospital	738 (59.18%)
Tertiary hospital	229 (18.37%)
Secondary hospital	218 (17.48%)
Primary hospital	62 (4.97%)
Hospital types (*n*, %)	
Public hospital	1198 (96.07%)
Private hospital	49 (3.93%)
Professional titles (*n*, %)	
Chief physician	348 (27.91%)
Associate chief physician	372 (29.83%)
Attending physician	355 (28.47%)
Resident physician	172 (13.79%)

### 3.2 Dermatologists' Preferences for LLM Responses

To assess dermatologists' preferences for LLM outputs, five categories of clinical questions related to psoriasis, specifically concerning etiology, clinical presentation, differential diagnosis, treatment, and case study, were presented to five different LLMs: ChatGPT‐4o (Model 1), Kimi.ai (Model 2), Doubao (Model 3), ZuoYiGPT (Model 4), and Lingyi‐agent (Model 5).

**Figure 1:**
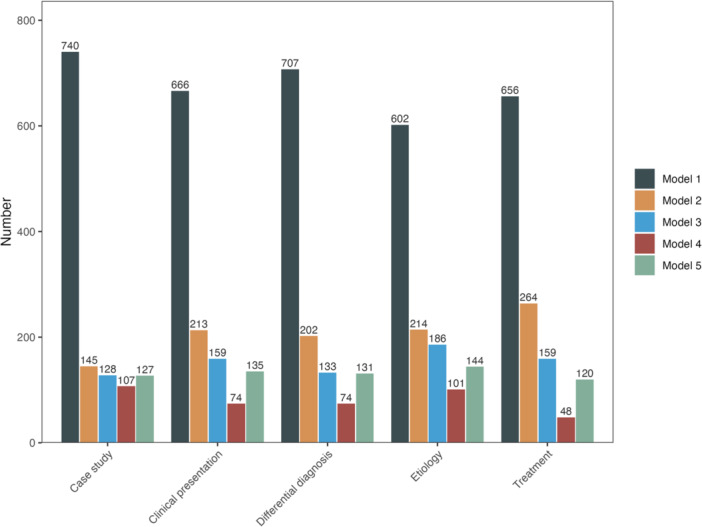
Dermatologists' preferences for responses generated by five large language models across different categories of clinical questions. This bar chart displays the total number of times each model was selected for five categories of clinical questions: case study, clinical presentation, differential diagnosis, etiology, and treatment. Model 1 was the most frequently preferred across all categories.

Model 1 was consistently the most preferred across categories of all clinical questions, receiving the highest number of selections for case study (*n* = 740), clinical presentation (*n* = 666), differential diagnosis (*n* = 707), etiology (*n* = 602), and treatment (*n* = 656) (Figure 1). Model 2 was the second most preferred, particularly for treatment (*n* = 264) and etiology (*n* = 214). In contrast, Models 3, 4, and 5 were selected less frequently, with Model 4 receiving the fewest votes overall (e.g., only 48 for treatment). These results underscore a clear and consistent preference for Model 1 across all clinical scenarios, with only minor variation in secondary preferences.

When the data were analyzed by professional title, a statistically significant difference in model preference was found solely for the differential diagnosis task (*χ* ^2^ = 21.13, *df* = 12, *p* = 0.0485). No significant differences were identified for the remaining four categories of clinical questions (all *p* > 0.05), indicating a general consistency in preferences across different professional ranks (Table 2).

**Table 2:** Differences in model selection by physician title (chi‐square test).

Question	*df*	Chi‐square	*p*‐value
Etiology	12	17.89	0.1192
Clinical presentation	12	8.47	0.7475
Differential diagnosis	12	21.13	0.0485
Treatment	12	3.60	0.9896
Case study	12	4.15	0.9805

Model preferences also did not show significant variation across hospital tiers. For all categories of clinical questions, etiology (*p* = 0.5230), clinical presentation (*p* = 0.1437), differential diagnosis (*p* = 0.6385), treatment (*p* = 0.6653), and case study (*p* = 0.4382), the *p*‐values were all greater than 0.05, suggesting a uniform pattern of model selection across different hospital tiers (Table 3).

**Table 3:** Differences in model selection by hospital tier (chi‐square test).

Question	*df*	Chi‐square	*p‐*value
Etiology	12	11.07	0.5230
Clinical presentation	12	17.16	0.1437
Differential diagnosis	12	9.74	0.6385
Treatment	12	9.44	0.6653
Case study	12	12.09	0.4382

### 3.3 Dermatologists' Evaluation Dimension Preferences

To assess how dermatologists evaluated LLM‐generated content, five quality dimensions were considered: accuracy, comprehensiveness, conciseness, logicality, and traceability. Participants were instructed to rate only one dimension as “very important,” designating their top priority.

**Figure 2:**
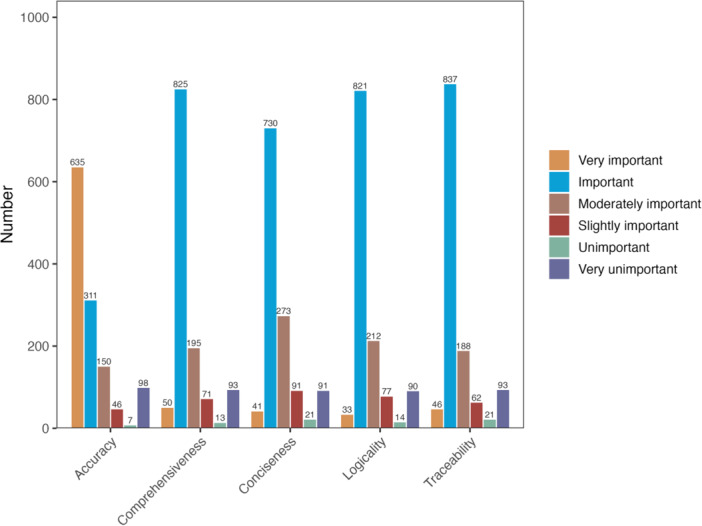
Importance ratings for five evaluation dimensions of LLM‐generated responses. This stacked bar chart illustrates the number of responses at each importance level—from “very important” to “very unimportant”—for the five evaluation dimensions: accuracy, comprehensiveness, conciseness, logicality, and traceability. While participants rated all five dimensions, they were allowed to designate only one as “very important.” Accuracy received the highest number of “very important” ratings, with “important” being the most common overall rating.

Accuracy was rated as the most important attribute, receiving the highest number of “very important” ratings (*n* = 635). In contrast, comprehensiveness (*n* = 50), traceability (*n* = 46), conciseness (*n* = 41), and logicality (*n* = 33) were selected less frequently. However, most participants rated the remaining dimensions as at least “important,” with traceability (*n* = 837), comprehensiveness (*n* = 825), and logicality (*n *= 821) receiving particularly high importance ratings, suggesting broad recognition of their relevance (Figure 2). Responses indicating “unimportant” or “very unimportant” were rare, highlighting the perceived significance of all five dimensions in evaluating clinical information.

**Figure 3:**
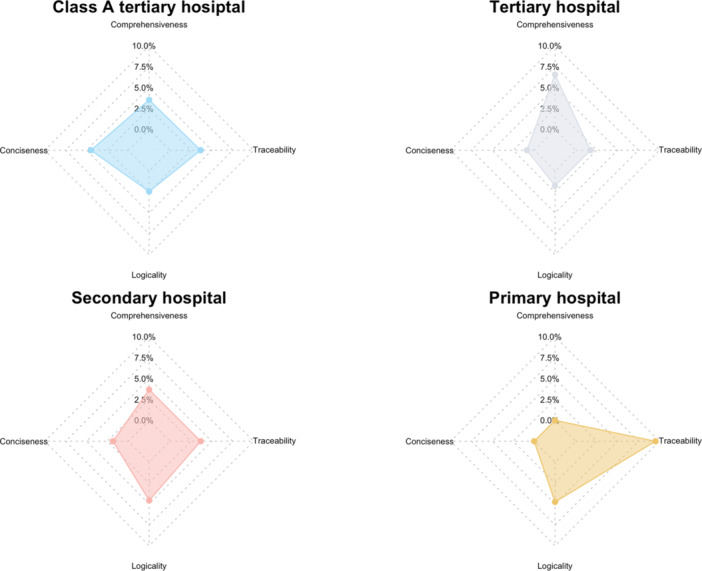
Proportion of “very important” ratings by hospital tier. Radar charts depicting the percentage of “very important” responses assigned to four evaluation dimensions—comprehensiveness, conciseness, logicality, and traceability—by dermatologists from different hospital tiers. Accuracy is excluded from the analysis, as it dominated across all groups.

Dermatologists from primary hospitals showed a distinct preference for traceability, assigning it a notably higher proportion of “very important” ratings compared with other dimensions. In contrast, dermatologists from Class A tertiary, tertiary, and secondary hospitals demonstrated a more balanced distribution of preferences across conciseness, traceability, and logicality, with comprehensiveness consistently ranked lower across all groups (Figure 3). A chi‐square test showed a significant association between hospital tier and the dimension considered most important by dermatologists (*χ* ^2^ = 27.667, *df* = 9, *p* = 0.0011), indicating that hospital tiers affect dermatologists' priorities in evaluation.

**Figure 4:**
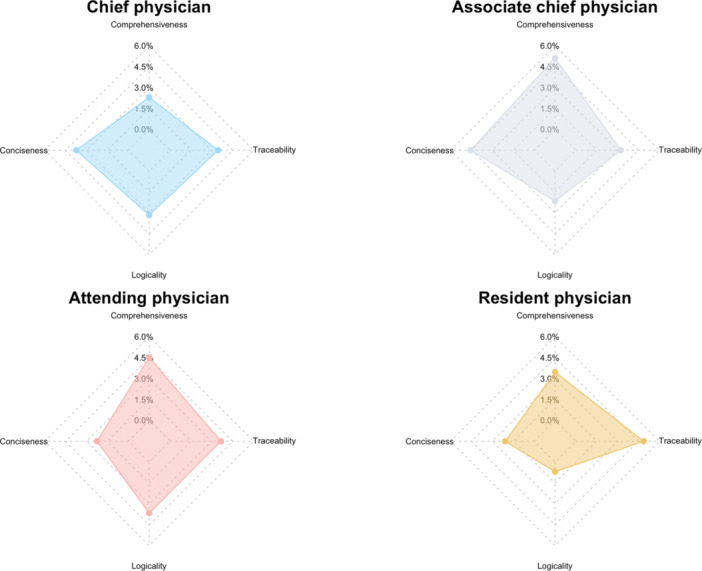
Distribution of “very important” ratings by physician title. Radar charts illustrating the distribution of “very important” ratings across four evaluation dimensions—comprehensiveness, conciseness, logicality, and traceability—among dermatologists of different professional titles. Accuracy is excluded, as it was the most frequently rated dimension across all groups.

Preferences for the four nondominant dimensions were relatively evenly distributed across different professional titles. Associate chief physicians tended to favor comprehensiveness and conciseness, while resident physicians more frequently prioritized traceability. Chief and attending physicians showed a more balanced distribution across all four dimensions. However, these differences were not statistically significant (*χ* ^2^ = 13.88, *df* = 9, *p* = 0.127), suggesting that professional title did not significantly affect dimension prioritization (Figure 4).

## 4 Discussion

This study is one of the first large‐scale investigations into dermatologists' perceptions and evaluations of LLMs in clinical dermatology in China. Among the five models assessed, ChatGPT‐4o (Model 1) was consistently preferred across all categories of clinical questions, including etiology, clinical presentation, differential diagnosis, treatment, and case study. This preference was consistent across different hospital tiers and professional titles, suggesting widespread acceptance and favorable usability of this model.

When evaluating the content quality of LLM‐generated responses, accuracy was the top priority for most participants. However, other dimensions—traceability, logicality, and comprehensiveness—were also rated highly as “important,” reflecting their recognized relevance in clinical decision‐making. Dermatologists in primary hospitals were more likely to rank traceability as the most important attribute, indicating context‐dependent differences in informational priorities based on the hospital tiers.

A qualitative review of the model outputs revealed distinct strengths and weaknesses. Model 2 (Kimi.ai) performed well in several key dimensions, including accuracy, comprehensiveness, logical coherence, and source traceability. It was also the only model to include explicit citations, aligning with the need for transparency and verifiability in clinical decision support. Model 1 (ChatGPT‐4o) and Model 3 (Doubao) each demonstrated specific advantages: Model 1 offered clear and concise responses, while Model 3 provided more detailed information. However, both models lacked explicit references, limiting the traceability of their content and potentially reducing their reliability in clinical contexts. Model 5 (Lingyi‐agent) appeared suitable for general information retrieval in low‐risk situations but lacked sufficient professional depth for clinical use. In contrast, Model 4 (ZuoYiGPT) showed notable issues with content accuracy and logical consistency, with some responses raising concerns about factual correctness.

Despite the relatively well‐structured and traceable responses from Model 2, it was chosen less frequently than Model 1. This discrepancy between content quality and user preference was consistent with patterns observed in medical AI research [13]. One likely explanation is the differences in response style and readability. Model 1′s responses were phrased in a conversational, fluent manner, resembling human‐written text. Its concise and accessible language made it easier for participants to process, particularly in the time‐constrained setting of a web‐based survey. In contrast, Model 2′s responses were longer, more technical, and included formal citations. While these features enhance scientific rigor, they may have increased perceived complexity, thereby reducing readability and user preference.

Additionally, since no explicit evaluation rubric was provided in the survey, participants likely relied on intuitive impressions when selecting their preferred responses. Thus, factors such as fluency, clarity, and perceived credibility may have been prioritized over comprehensive content or verifiability. Consequently, user preferences in this context may reflect subjective judgments about readability and linguistic naturalness rather than a systematic assessment of medical accuracy.

These findings were consistent with a well‐established insight in LLM user experience research: outputs that are easier to read and more naturally phrased often receive more favorable responses, even if they are technically simpler. For clinical LLMs to be effectively integrated into practice, developers must consider not only the content′s accuracy but also how it is presented and experienced by end users.

Previous studies evaluating LLMs in medicine have typically relied on expert scoring or objective benchmarks, with relatively few focusing on user‐centered assessments. For instance, Goktas et al. [14] demonstrated that ChatGPT showed high diagnostic concordance with dermatology specialists. Additionally, a recent cross‐cultural evaluation of LLMs in traditional Chinese medicine showed that general‐purpose models like GPT‐4o could generate clinically relevant diagnostic and treatment recommendations comparable to those made by professional acupuncturists. This highlights the growing role of LLMs in culturally specific healthcare contexts [15]. Our findings support the consistent preference for GPT‐based models in clinical settings. However, we expand on this understanding by showing that dermatologists assess content quality based on multiple dimensions, with a particular emphasis on traceability and logical coherence—especially in resource‐limited environments.

Similar to studies in other specialties, such as oncology [16], accuracy was the most prioritized criterion. However, preferences for content traceability and structure varied depending on institutional context and access to information, underscoring the situational nature of clinical AI evaluation.

This study has several limitations. First, the use of convenience sampling may affect the generalizability of the findings. Participants were mainly recruited through dermatology‐related WeChat groups, which may not fully represent the broader dermatology workforce in China. Specifically, the sample was skewed toward physicians from tertiary hospitals (more than half of participants), with dermatologists from primary hospitals underrepresented. This imbalance may limit the applicability of the findings to different hospital tiers and regional healthcare settings. Second, while mechanisms were implemented to prevent duplicate submissions, participant identities were self‐reported, indicating that some misclassification cannot be ruled out. Third, this study focused exclusively on psoriasis, suggesting that the findings may not be applicable to other dermatological conditions. The perceived utility of LLMs may vary depending on disease type and complexity. Fourth, participants were required to select only one “very important” dimension from a predefined list. This design aimed to simplify the response process, reduce participant burden, and prioritize the most critical factor in clinical evaluation. However, this approach may not fully capture the multidimensional nature of real‐world clinical decision‐making, where accuracy, traceability, logicality, and other factors often interact. In addition, the five categories of clinical questions included in the survey cannot comprehensively represent the range of dermatological practice. Scenarios such as multimodal tasks integrating imaging and pathology or longitudinal tasks like drug therapy monitoring were not assessed. These omissions may limit the generalizability of our findings to broader clinical contexts. Finally, this study focused on subjective preferences and perceptions without evaluating the actual clinical impact of LLM use on diagnostic accuracy or patient outcomes. These aspects warrant further investigation in future studies. Future research should incorporate objective quantitative metrics, such as readability scores, completion time, and physician satisfaction ratings, to provide more robust and reproducible evidence on the comparative advantages of different LLMs.

## 5 Conclusions

In this nationwide cross‐sectional survey, Chinese dermatologists demonstrated a consistent preference for ChatGPT‐4o over domestic LLMs when addressing clinical questions related to psoriasis. While accuracy was the most highly valued content attribute, traceability and logicality were also deemed critical, particularly by clinicians in primary hospitals. As LLMs continue to evolve and integrate into healthcare workflows, aligning technical performance with the specific preferences and practical needs of clinicians will be crucial for ensuring the reliable and successful adoption of these tools in clinical practice.

## Author Contributions

**Jungang Yang:** conceptualization, investigation, methodology, data curation, formal analysis, visualization, writing – original draft. **Jingkai Xu:** conceptualization, data curation, supervision, writing – review and editing. **Xuejiao Song:** project administration, data curation. **Chengxu Li:** project administration, data curation. **Lili Chen:** data curation. **Lingbo Bi:** data curation. **Tingting Jiang:** data curation. **Xianbo Zuo:** writing – review and editing, supervision. **Yong Cui:** writing – review and editing, supervision.

## Ethics Statement

The study protocol was approved by the Ethics Committee of China‐Japan Friendship Hospital (2024‐KY‐286), and it was compliant with the Helsinki Declaration of 1975, as revised in 2024.

## Consent

Participants received study information before the survey, and completion of the questionnaire implied informed consent. No identifiable personal data were collected.

## Conflicts of Interest

The authors declare no conflicts of interest.

## Supporting information

PROOF‐Data S1.

## Data Availability

The data that support the findings of this study are available from the corresponding author upon reasonable request.
