# Supplementary material for: Preferences of Chinese Dermatologists for Large Language Model Responses in Clinical Psoriasis Scenarios: A Nationwide Cross‐Sectional Survey in China
Source: Health Care Sci. 2026 Feb 11;5(1):40–8. doi: 10.1002/hcs2.70057 (PMC12946707; doi:10.1002/hcs2.70057)
Supplement: Supplementary file 1 — PROOF‐Data S1. [file HCS2-5-40-s001.docx]

The questionnaire of survey

| **Question** | **Choice** |
| --- | --- |
| Part 1: Basic information | |
| Your gender | ○ Male  ○ Female |
| Your age | (Fill in the blanket) |
| Grade of your hospital | ○ Class A tertiary hospital  ○ Tertiary hospital  ○ Secondary hospital  ○ Primary hospital |
| Type of your hospital | ○ Public hospital  ○ Private hospital |
| Your department | ○ Dermatology  ○ Others |
| Your title | ○ Chief physician  ○ Associate chief physician  ○ Attending physician  ○ Resident physician |
| Part 2: Preference of large language models’ answer | |
| Which of the following five large language model responses to the question about the etiology of psoriasis vulgaris do you prefer? | ○ Model 1  The exact etiology of psoriasis is not yet fully understood, but studies have shown that its occurrence is related to genetic predisposition, immune system abnormalities, and environmental factors. Specifically, the main aspects include:  1.Genetic Factors  Genetic susceptibility is one of the most important factors for psoriasis. About 30% of psoriasis patients have a family history, indicating that heredity plays an important role in the development of the disease. The genetic basis of psoriasis is usually considered to be the result of the combined action of multiple genes, particularly those related to the immune system. Studies have shown that specific human leukocyte antigen (HLA) genes—especially HLA-Cw6—are closely associated with the occurrence of psoriasis.  2. Immune System Abnormalities  Psoriasis is a chronic, immune-mediated inflammatory skin disease, primarily triggered by the abnormal activation of T cells in the skin. Dendritic cells and other immune cells participate in inflammatory responses. These immune cells release pro-inflammatory cytokines such as TNF-α (tumor necrosis factor alpha) and IL-17 (interleukin-17), leading to abnormal proliferation of keratinocytes and impaired skin barrier function, thereby forming the characteristic lesions of psoriasis vulgaris.  3. Environmental Factors  Many environmental factors can induce or aggravate psoriasis. Common environmental triggers include:  Infection: Streptococcal infection, especially of the respiratory tract, is a common trigger of psoriasis in children and adolescents.  Medications: Certain drugs may induce or exacerbate psoriasis, such as beta-blockers, lithium, and antimalarials.  Skin Trauma: Skin injury, surgery, or sunburn can induce psoriasis lesions at the site of trauma, known as the Koebner phenomenon.  Stress: Psychological stress and emotional fluctuations are common triggers, and many patients experience exacerbations during significant emotional events.  Smoking and Alcohol: Smoking and excessive alcohol consumption are considered risk factors for psoriasis and may worsen the condition or trigger disease onset.  4. Endocrine Factors  Psoriasis may worsen during certain endocrine changes (such as puberty or menopause). Female patients may experience aggravation during pregnancy or while taking hormonal medications, with possible symptom fluctuations.  5. Metabolic and Systemic Diseases  Psoriasis is associated with some metabolic diseases (such as obesity and diabetes) and cardiovascular diseases. These conditions may affect the immune system or promote chronic inflammation, indirectly exacerbating psoriasis severity.  Summary  The onset of psoriasis vulgaris results from the combined effects of multiple factors, among which genetic susceptibility and immune system abnormalities are the main causes. Environmental factors—such as infection, trauma, and stress—also play important roles. The specific triggers vary among individuals, so personalized treatment and management strategies are essential.  ○ Model 2  The exact cause of psoriasis vulgaris is still unclear, but it is currently believed to result from the combined effects of genetic, immune, and environmental factors*.  Genetic Factors  Psoriasis is a polygenic hereditary disease with a genetic predisposition. If both parents have psoriasis, the risk of their child developing the disease is 41%; if only one parent has psoriasis, the child’s risk is 14%*.  About 20% of psoriasis patients have a family history of the disease*.  Immune Factors  In psoriasis patients, skin lesions show infiltration of lymphocytes and mononuclear cells, especially T lymphocytes and dendritic cells, indicating that the immune system participates in the onset and progression of psoriasis*.  It is currently believed that the IL-23/IL-17A inflammatory pathway plays a major role in the pathogenesis of psoriasis*.  Environmental Factors  Infection: Infections such as streptococcal, staphylococcal, and fungal infections are known triggers for psoriasis*.  Psychological Factors: Emotional stress, stressful events, and psychological trauma can induce or exacerbate psoriasis*.  Trauma: Physical injuries, scratches, or sunburn may lead to psoriasis lesions (Koebner phenomenon)*.  Medication: Certain drugs can trigger psoriasis, including some antihypertensive drugs (e.g., β-blockers) and psychiatric medications (e.g., lithium)*.  Lifestyle Factors: Smoking and excessive alcohol consumption can impair immune cell function, increasing the risk of psoriasis. In addition, obesity is also a risk factor for psoriasis*.  Note: the asterisk * denotes a source that can be accessed online for further information.  ○ Model 3  Psoriasis vulgaris is a common chronic inflammatory skin disease. Its causes are relatively complex and mainly include the following aspects:  I. Genetic Factors  Psoriasis has a clear hereditary tendency. If there are psoriasis patients in a family, other family members are at a significantly higher risk of developing the disease. Multiple studies show that the concordance rate of psoriasis in monozygotic twins is about 70%, while that in dizygotic twins is about 20%. This suggests that genetic factors play a key role in the pathogenesis of psoriasis, possibly influencing processes such as epidermal cell proliferation, differentiation, and immune response.  II. Immune Factors  The immune system plays a central role in the development of psoriasis. T lymphocytes are particularly important in initiating and maintaining the disease. In psoriatic lesions, T cells become abnormally activated and release inflammatory cytokines, such as tumor necrosis factor-alpha (TNF-α), interleukin-17 (IL-17), and interleukin-23 (IL-23).  IL-17 can promote keratinocyte proliferation and excessive differentiation, while also recruiting inflammatory cells into the lesion, thereby amplifying the inflammatory response. The interactions between immune cells and keratinocytes form a complex inflammatory network that drives the progression of psoriasis.  III. Environmental Factors  1. Infectious Factors:  Bacterial infections, especially streptococcal infections, are closely associated with the onset and exacerbation of psoriasis. For instance, children who experience streptococcal throat infections may develop guttate psoriasis, possibly due to molecular mimicry between bacterial antigens and self-antigens, leading to immune activation and skin inflammation.  2. Psychological Factors:  Chronic stress, anxiety, and tension can disrupt neuroendocrine-immune regulation. For example, stress activates the hypothalamic-pituitary-adrenal (HPA) axis, increasing the secretion of stress-related hormones. These changes can affect immune homeostasis, impair immune tolerance, and increase susceptibility to psoriasis.  3. Drug-Induced Factors:  Certain medications may trigger or exacerbate psoriasis. Examples include β-blockers (such as propranolol), antimalarial drugs, and lithium. These drugs can influence intracellular signaling or immune modulation, thereby promoting or worsening psoriatic inflammation.  IV. Skin Barrier Dysfunction  The skin barrier mainly consists of the stratum corneum and lipids. When the barrier is damaged, external irritants and allergens can more easily penetrate the skin, activating immune and inflammatory pathways. Psoriasis patients often exhibit increased epidermal turnover and barrier dysfunction, which further aggravates inflammation and disease progression. Impaired skin barrier function is both a consequence and a contributing factor to psoriasis.  ○ Model 4  The exact causes of psoriasis vulgaris are not yet fully understood. It is currently believed to be related to genetic, infectious, immune, neuropsychological, endocrine, and environmental factors.  1. Genetic Factors  Psoriasis vulgaris has a familial tendency, with 10%–30% of patients having a family history. The heritability is estimated at 44%–88%. The development of psoriasis vulgaris is polygenic and also influenced by environmental factors.  2. Infection  Many studies have demonstrated through humoral immunity (anti-streptococcal antibodies), cellular immunity (peripheral blood and lesional T cells), and bacterial culture and treatment that streptococcal infections are closely associated with the onset of psoriasis vulgaris.  In patients with psoriasis vulgaris, Staphylococcus aureus infection can also exacerbate the disease.  3. Immune Abnormalities  A large body of research indicates that psoriasis vulgaris is an immune-mediated inflammatory skin disease, and its pathogenesis is closely related to T-cell dysregulation.  4. Endocrine Factors  Some female patients experience improvement or remission of psoriasis during pregnancy, while others may worsen after delivery, indicating a role of hormonal regulation.  5. Psychological Factors  Psychological factors are associated with the onset of psoriasis vulgaris. Emotional stress or psychological pressure may trigger or exacerbate psoriatic symptoms.  6. Other Factors  External factors such as cold weather, humid environments, infections, surgery, trauma, and dry climates may also trigger or worsen psoriasis vulgaris.  ○ Model 5  The causes of psoriasis vulgaris mainly include the following aspects:  1. Immune Factors  Various immune cells such as T cells, dendritic cells, neutrophils, and keratinocytes participate in psoriasis pathogenesis by releasing cytokines including tumor necrosis factor-alpha (TNF-α), interferon-gamma (IFN-γ), interleukin-17 (IL-17), and interleukin-22 (IL-22). These cytokines trigger psoriatic-specific changes such as neutrophil infiltration and angiogenesis. Activated dendritic cells produce IL-1, IL-23, and IL-12, which induce or enhance the activation and proliferation of Th1, Th17, and Th22 cells. These cells secrete cytokines including TNF-α, IL-17, and IL-22, which in turn stimulate keratinocyte hyperproliferation and differentiation-related genes, forming a self-perpetuating inflammatory loop. Among these, IL-23 and Th17 cells play central roles — IL-23 promotes the production of IL-17 by Th17 cells, and IL-17A is considered most closely related to the pathogenesis of psoriasis.*  2. Genetic and Immune Response Abnormalities  The exact pathogenic genes and mechanisms of psoriasis vulgaris remain unclear, but genetic predisposition and abnormal immune responses play major roles. Chinese researchers have discovered that mutations in the IL36RN gene (interleukin-36 receptor antagonist) may be key causative factors in generalized pustular psoriasis (GPP). The mutation rate of IL36RN in GPP patients with psoriasis vulgaris is around 71%, while in non-psoriatic GPP patients it is 38%. This suggests that IL36RN mutations may contribute significantly to psoriasis susceptibility.*  3. Environmental Factors  The onset and progression of psoriasis are also influenced by environmental factors such as infections, stress, medications, alcohol consumption, and smoking. These factors can disrupt immune homeostasis and stability, thereby aggravating psoriatic symptoms and promoting disease progression.*  In summary, psoriasis vulgaris is a complex, multifactorial disease involving immune dysregulation, genetic susceptibility, and environmental influences.  Note: the asterisk * denotes a source that can be accessed online for further information. |
| Which of the following five large language model responses to the question about the clinical presentation of psoriasis vulgaris do you prefer? | ○ Model 1  Psoriasis vulgaris is the most common type of psoriasis. Its clinical features are distinctive, typically presenting as chronic, relapsing inflammatory skin lesions. The main clinical manifestations can be summarized in the following six aspects:  1. Cutaneous Features  Psoriasis vulgaris typically presents with several characteristic skin findings:  Erythematous Plaques with Scales: The hallmark lesion is a well-demarcated erythematous plaque covered with silvery-white scales. The base appears bright red, and the surface scales are easily detached.  Auspitz Sign: When the scales are gently scraped off, small pinpoint bleeding points appear on the underlying surface, known as the Auspitz sign.  Koebner Phenomenon: New lesions may appear along sites of trauma, scratching, or irritation, a response referred to as the Koebner phenomenon.  Candle-Grease Sign: When scraping the scales, a phenomenon similar to candle wax shavings appears.  Distribution: Lesions are commonly found on the scalp, extensor surfaces of the limbs, elbows, knees, and lower back. In severe cases, lesions can merge into large areas of affected skin.  2. Typical Clinical Presentations  Guttate Psoriasis: This form appears as numerous small, drop-like papules covered with thin, silvery scales, commonly occurring after streptococcal infection and seen more often in children and adolescents.  Plaque Psoriasis: The most common form, characterized by erythematous plaques of varying sizes covered with thick silvery scales, often located on scalp, elbows, and knees.  Flexural Psoriasis: Involves intertriginous areas such as axillae, groin, and inframammary folds. The lesions are bright red, shiny, and usually lack scales due to moisture and friction.  Nail Psoriasis: Manifests as pitting, subungual hyperkeratosis, onycholysis, and oil-drop discoloration. Approximately 30–50% of psoriasis patients exhibit nail involvement.  Palmoplantar Psoriasis: Commonly presents with thickened plaques, fissures, and sometimes painful cracking or bleeding.  Erythrodermic Psoriasis: A rare but severe generalized form involving widespread erythema, scaling, and systemic symptoms such as fever and malaise.  3. Associated Symptoms  Pruritus: Most patients experience itching of varying degrees, though sometimes mild.  Skin Dryness: Affected areas often appear dry, rough, and prone to cracking or bleeding.  Nail Changes: About 30–50% of patients show nail pitting, onycholysis, or yellow-brown discoloration.  Systemic Symptoms: Some severe cases may show fever, fatigue, or malaise, though mild psoriasis generally presents only with cutaneous findings.  4. Chronicity and Recurrence  Psoriasis vulgaris tends to be chronic and relapsing, often going through cyclic flare-ups and remissions. Many patients experience seasonal variation, with exacerbations in winter and improvement in summer. Even during remission, residual pigmentation or mild scaling may persist.  5. Disease Course  Early Stage: Small red papules gradually enlarge and coalesce, forming well-defined plaques covered with scales.  Progressive Stage: Lesions continue to expand, with active inflammatory borders and increased scaling.  Stable Stage: The disease stabilizes, with persistent plaques and little change.  Regressive Stage: The scales gradually disappear, leaving post-inflammatory hyperpigmentation or hypopigmentation.  6. Histopathological Changes  Histologically, psoriasis vulgaris shows: Epidermal hyperplasia with elongated rete ridges; Parakeratosis and absent granular layer; Munro microabscesses (collections of neutrophils within the stratum corneum); Dilated dermal capillaries and inflammatory cell infiltration.  These histological features correlate with epidermal proliferation, immune activation, and inflammatory response.  Summary  Psoriasis vulgaris is a chronic inflammatory skin disease characterized by erythematous, scaly plaques with a chronic relapsing course. It often involves itching, seasonal variation, nail changes, and systemic immune abnormalities. Understanding its clinical and histopathological features is essential for accurate diagnosis and individualized treatment.  ○ Model 2  Psoriasis vulgaris is the most common type of psoriasis. Its clinical manifestations can be summarized into the following aspects:  Skin Manifestations  Erythematous Plaques: The skin shows well-demarcated red plaques of varying size and shape, often surrounded by inflammatory redness and mild swelling or thickening.  Silvery-White Scales: The surface of the plaques is covered with multiple layers of silvery-white scales that are easily detached. When the scales are scraped off, a semi-transparent shiny film appears beneath — this is known as the membranous phenomenon.  Pinpoint Bleeding (Auspitz Sign): When the thin membrane is scraped away, small pinpoint bleeding spots appear underneath, known as the Auspitz sign, one of the hallmark diagnostic features of psoriasis vulgaris*.  Distribution Pattern: Lesions most commonly occur on extensor surfaces of the limbs (such as elbows and knees), as well as the sacral and scalp regions, often showing symmetrical distribution*.  Other Manifestations  Itching: Some patients may experience mild to moderate itching*.  Skin Dryness and Cracking: The skin may become dry, rough, and fissured, sometimes even bleeding.  Scalp Involvement: Psoriasis on the scalp presents as thick scaly plaques that may cover the entire scalp*.  Nail Changes: May present as nail pitting, onycholysis (nail detachment), or subungual hyperkeratosis (thickening beneath the nail)*.  Disease Stages  Psoriasis vulgaris typically progresses through three stages:  Progressive Stage: New lesions continue to appear and expand, with active inflammation and often positive Koebner phenomenon (new lesions appearing at sites of trauma)*.  Stationary Stage: Lesions stabilize with no new eruptions, inflammation subsides, and scaling becomes lighter*.  Regressive Stage: Lesions begin to flatten and resolve, with scales shedding, and post-inflammatory pigmentation (light or dark spots) remaining*.  Note: the asterisk * denotes a source that can be accessed online for further information.  ○ Model 3  Clinical Manifestations of Psoriasis Vulgaris  I. Skin Lesions  1. Scales  Typical psoriatic skin lesions are covered by a large number of scales, which are silvery-white, resembling mica flakes or dandruff-like patches on the skin surface. The scales are loosely attached and easily shed. After the scales are removed, a thin, transparent, shiny membrane appears underneath — known as the “film phenomenon”. If this membrane is scraped away, pinpoint bleeding occurs, called the “Auspitz sign.” These characteristic findings can usually be seen on areas such as the elbows, knees, and shins when gently scraped.  2. Erythema  Erythema is another major feature of psoriasis vulgaris. The lesions appear as well-demarcated red patches or plaques of varying sizes and shapes. In the early stage, the redness is light pink, gradually becoming dark red or bright red as the condition progresses. Erythematous lesions may appear in different forms, including guttate (drop-like), coin-shaped, geographic (map-like), or annular (ring-shaped) patterns.  Guttate psoriasis is common in children and adolescents, usually appearing at disease onset as numerous small, scattered, red papules of 3–10 mm in diameter. The surface is covered with fine scales resembling dew drops on a leaf. These lesions may merge into larger plaques with irregular shapes, similar to geographic maps.  3. Papules  In addition to plaques, papules may also appear — small, elevated solid lesions ranging from millet-sized to pea-sized, usually red or pink. The surface is covered with silvery scales, which may coalesce into larger plaques or remain isolated. These are often seen on areas such as the scalp, elbows, and knees.  II. Common Sites of Involvement  1. Scalp  The scalp is a very common site for psoriasis vulgaris. Lesions appear as well-defined erythematous plaques with thick, silvery scales, sometimes extending beyond the hairline, forming what is known as a “crown-like pattern.” This presentation is often referred to as sebopsoriasis or psoriatic dandruff. When severe, scales may cover the entire scalp.  2. Extensor Surfaces (Elbows and Knees)  Lesions often appear symmetrically on the elbows and knees. Due to friction and external irritation, scales here are typically thicker and more persistent. These are among the most common sites of psoriasis vulgaris.  3. Back  The back is another area frequently affected. Lesions appear as red plaques with thick scales, which can be itchy or uncomfortable, especially due to friction from clothing.  4. Sacral Region  Lesions may also occur in the sacral or lumbar region, appearing as erythematous plaques with scaling. Because of local pressure and limited blood flow, lesions here tend to persist and heal slowly.  III. Subjective Symptoms  1. Itching  Most patients experience some degree of itching, though the severity varies. Mild cases present only slight itchiness, while severe cases may experience intense itching, affecting sleep and daily life. The itching is mainly due to skin inflammation and nerve hypersensitivity.  2. Burning Sensation  During the acute stage or when lesions are rapidly progressing, some patients may feel a burning or stinging sensation, especially on inflamed areas. This is more likely to occur in autumn and winter, when the air is dry and skin loses moisture easily, making irritation more noticeable.  ○ Model 4  Psoriasis vulgaris can be divided into three stages according to disease progression: progressive stage, stationary stage, and regressive stage.  1. Progressive stage  New lesions continue to appear, and existing lesions gradually enlarge. Physical stimulation such as needling, scratching, friction, or surgery can induce new psoriatic lesions, which is called the Koebner phenomenon. Inflammation is obvious, scales are thick and easy to remove, and removal often causes pinpoint bleeding. New lesions may appear around existing ones, forming a characteristic crown-like pattern.  2. Stationary stage  No new lesions appear, existing lesions remain stable without changes, inflammation is reduced, and scales are decreased.  3. Regressive stage  Lesions shrink or flatten, inflammation basically subsides, and pigmentation may remain or fade. Psoriasis vulgaris usually occurs on the scalp and the extensor sides of the limbs, especially the elbows and knees, and is often symmetrical. The lesions are thick, covered with silvery-white scales that are easy to peel off. After removing the scales, a thin, transparent film (the film phenomenon) can be seen, and gently scraping it may cause small bleeding points (the Auspitz sign). The lesions vary in shape, appearing as guttate, coin-shaped, geographic, or annular types. Some patients may feel itching. Psoriasis vulgaris usually has a slow, chronic course and may improve spontaneously.  ○ Model 5  The clinical manifestations of psoriasis vulgaris mainly include the following aspects:  1. Basic lesions: Initially, red papules or plaques of millet to soybean size appear, covered with silvery white scales. In acute cases, the scales are fewer; in chronic lesions, the scales are more. The scales are easily detached by scraping, showing the phenomena of wax-drop, thin film, and punctate bleeding. Some patients may have itching. Due to different lesion sites, the clinical manifestations vary.  2. Scalp lesions: Scalp psoriasis may appear.  3. Finger (toe) and nail changes: Depressions like needle pricks can be seen on the nail surface, and there may be “oil drop”-like spots on the nails.  4. Mucosal lesions: Red patches with clear boundaries and without scales may be seen on mucous membranes. Silver-white scales can appear on the lips. Grayish-white or white ring-shaped patches may be seen on the buccal mucosa and upper lip. A few cases involve the eyes, such as eyelids and conjunctiva.*  5. Plaque psoriasis: This is the most common type of psoriasis, accounting for 80%-90% of cases. The lesions usually occur on the scalp, lumbosacral area, elbows, knees, and the extensor sides of the limbs. Typical lesions present as dark red plaques or infiltrative erythematous patches with clear boundaries, with or without itching, covered by thick silver-white scales. When the scales are lightly scraped, they fall off easily, showing the phenomena of wax-drop, thin film, and punctate bleeding (Auspitz sign). These features are diagnostic for psoriasis vulgaris. According to disease progression, psoriasis vulgaris can be divided into three stages: progressive stage, stationary stage, and regressive stage.*  6. TCM syndrome differentiation criteria: According to the lesion site, clinical manifestations vary—scalp lesions may show dandruff-like scales; finger (toe) nails may show needle-like depressions or detachment, loss of luster; mucous membranes may show red patches with clear boundaries and without scales.*  In summary, the clinical manifestations of psoriasis vulgaris are diverse, involving multiple body parts and various symptoms.  Note: the asterisk * denotes a source that can be accessed online for further information. |
| Which of the following five large language model responses to the question about the differential diagnosis of psoriasis vulgaris do you prefer? | ○ Model 1  The clinical manifestations of psoriasis vulgaris may resemble several other skin diseases, so it is important to perform differential diagnosis during diagnosis. The following are the differential diagnoses of psoriasis vulgaris:  1. Seborrheic dermatitis  Clinical manifestations: Seborrheic dermatitis often occurs on the scalp, face, chest, and back, showing erythema with greasy scales, itching, and yellowish scales.  Diagnostic points: Seborrheic dermatitis lesions are mostly light red with greasy yellow scales, while psoriasis vulgaris has thicker, silvery-white scales. Seborrheic dermatitis has fuzzy borders, while psoriasis vulgaris has clearly demarcated lesions.  2. Eczema (atopic dermatitis)  Clinical manifestations: Eczema lesions are erythematous, edematous, exudative, and oozing, accompanied by severe itching and scaling.  Diagnostic points: Eczema often has multiple stages of lesions, such as papules, vesicles, and crusts, and lacks silvery scales. Psoriasis vulgaris, however, has thick, dry scales without exudation, with a chronic and relapsing course.  3. Pityriasis rosea  Clinical manifestations: Pityriasis rosea begins as a single herald patch, later followed by multiple oval red patches with scaling along the cleavage lines of the trunk, forming a Christmas tree-like pattern.  Diagnostic points: Pityriasis rosea lesions are mostly oval, with thin scales and mild inflammation, and resolve within about 6–8 weeks, while psoriasis vulgaris tends to be chronic and recurrent.  4. Tinea corporis  Clinical manifestations: Tinea corporis usually appears as a round or ring-shaped red patch with scaling, often forming a ring-like pattern as the lesion expands.  Diagnostic points: Tinea corporis is typically annular with a raised active border and central clearing. In addition, fungi can be found in the scales by microscopic examination, which is diagnostic for tinea corporis.  5. Drug eruption  Clinical manifestations: Drug eruptions vary widely in appearance, including macules, papules, erythema, and pustules. Certain medications may trigger psoriasis-like eruptions.  Diagnostic points: Drug eruptions generally have an acute onset and a clear history of medication use. After discontinuation of the offending drug, symptoms usually resolve, whereas psoriasis vulgaris tends to persist and relapse.  6. Chronic lupus erythematosus (such as discoid lupus erythematosus)  Clinical manifestations: Lesions are red or purplish-red plaques with follicular plugging, slight scaling, and sometimes atrophy or scarring, mainly occurring on the face and scalp.  Diagnostic points: Discoid lupus erythematosus lesions have clear borders with follicular keratotic plugs and atrophy in the center, while psoriasis vulgaris lacks such changes and shows thicker scales.  7. Secondary syphilis  Clinical manifestations: Secondary syphilis may present with widespread erythematous papules or plaques, sometimes with systemic symptoms such as fever and fatigue.  Diagnostic points: Secondary syphilis can be confirmed by serological testing. Psoriasis vulgaris lesions are not contagious and lack systemic syphilitic features.  8. Pityriasis rubra pilaris  Clinical manifestations: Pityriasis rubra pilaris presents with red-orange patches with follicular keratotic papules, later merging into large patches with scaling, often accompanied by palmoplantar keratoderma.  Diagnostic points: The color of pityriasis rubra pilaris lesions is orange-red, often involving the entire body with islands of normal skin. Psoriasis vulgaris shows silvery-white scales and a slower progression.  9. Erythroderma  Clinical manifestations: Erythroderma presents with widespread erythema and scaling, often accompanied by systemic symptoms such as fever, chills, and lymphadenopathy.  Diagnostic points: Erythroderma may develop from severe psoriasis vulgaris or other dermatoses and is accompanied by systemic signs, while psoriasis vulgaris itself has no significant systemic symptoms.  10. Neurodermatitis  Clinical manifestations: Neurodermatitis presents with localized thickened plaques due to chronic scratching, accompanied by severe itching, and the condition worsens with scratching.  Diagnostic points: Neurodermatitis lesions lack silvery-white scales and have unclear borders, while psoriasis vulgaris shows well-demarcated plaques with silvery scales.  Diagnostic methods: When diagnosing, physicians should make a comprehensive judgment based on medical history, clinical manifestations, skin lesions, and relevant auxiliary examinations (such as dermoscopy and pathological biopsy). Dermoscopy typically shows punctate bleeding points (Auspitz sign), silvery-white scales, and thickened epidermis (hyperkeratosis), all of which support the diagnosis of psoriasis vulgaris.  In summary, accurate diagnosis requires careful differentiation from other diseases to avoid misdiagnosis and delayed treatment.  ○ Model 2  The differential diagnosis of psoriasis vulgaris includes the following aspects:  Other types of psoriasis:  Pustular psoriasis: Manifested as sterile pustules, which can be generalized or localized. The pustules are obvious and often accompanied by fever, joint pain, and other systemic symptoms*.  Erythrodermic psoriasis: The whole body shows diffuse erythema and edema with numerous small scales and severe swelling. The normal skin is rare, and it may develop from psoriasis vulgaris*.  Psoriatic arthritis: In addition to skin lesions, there are also inflammatory joint diseases such as pain, swelling, pressure pain, stiffness, and movement restriction in joints and surrounding soft tissues*.  Other dermatoses:  Seborrheic dermatitis: Usually occurs in sebaceous gland-rich areas such as the face and upper body. Lesions are red patches with greasy scales*.  Tinea corporis: Lesions have scales and need fungal microscopy for diagnosis*.  Chronic eczema: Has the chronic characteristics of eczema with typical pathological findings different from psoriasis*.  Lichen planus: Purple polygonal papules with Wickham striae on the surface*.  Chronic cutaneous lupus erythematosus: Has atrophy, lesions are mostly located on the exposed parts*.  Pityriasis rosea: Has a herald patch, lesions are rose-colored with fine scales*.  Parapsoriasis: Isolated plaques, especially in cases that do not respond to treatment should be considered*.  Lichen simplex chronicus (neurodermatitis): Mainly presents with localized thickening and lichenification due to chronic scratching*.  Bowen’s disease: Erythematous plaques with thickened keratin layer and clustered small scales on the surface*.  Secondary syphilis: May show polymorphic rashes, including lesions similar to psoriasis*.  Scalp mycosis: Fungal infection of the scalp with hair loss*.  Note: the asterisk * denotes a source that can be accessed online for further information.  ○ Model 3  Psoriasis vulgaris needs to be differentiated from several skin diseases:  I. Seborrheic dermatitis  1. Clinical manifestations:  Seborrheic dermatitis often occurs on the scalp, eyebrows, nose wings, chest, and back, where sebaceous glands are abundant. Typical lesions are red patches covered with greasy yellow scales on the surface. For example, seborrheic dermatitis on the scalp has thinner, oilier scales, while psoriasis vulgaris has thicker, silvery-white scales with clear borders, thicker scales, wax-drop phenomenon, and Auspitz sign.  2. Pathogenesis difference:  Seborrheic dermatitis is mainly related to sebaceous gland secretion, Malassezia infection, and so on. The sebaceous secretion provides a good environment for Malassezia to survive, and the metabolic by-products of Malassezia stimulate the skin to produce inflammatory responses. Psoriasis vulgaris, however, is mainly caused by immune abnormalities and genetic factors, leading to excessive proliferation and inflammatory responses of epidermal cells.  II. Pityriasis rosea  1. Characteristics of skin lesions and distribution:  Pityriasis rosea usually starts with a single herald patch, generally round or oval, with a diameter of 2–5 cm, located on the trunk or proximal limbs. Within 1–2 weeks, multiple smaller daughter patches appear on the body, mostly distributed along the ribs or Langer’s lines. In contrast, psoriasis vulgaris lesions do not follow these specific lines of distribution, and the shape varies, such as coin-shaped or map-like lesions.  2. Features of scales and base:  The scales of pityriasis rosea are thinner than those of psoriasis vulgaris and lack the wax-drop phenomenon and Auspitz sign. Pityriasis rosea has self-limiting characteristics, generally resolving within 6–8 weeks, while psoriasis vulgaris is a chronic, relapsing disease.  III. Secondary psoriasis  1. Differences in clinical manifestations:  Secondary psoriasis includes guttate, pustular, plaque, and erythrodermic types. Among them, guttate secondary psoriasis lesions resemble guttate psoriasis in appearance but have thinner scales, are not easy to peel, and lack the typical wax-drop phenomenon and Auspitz sign. The plaque type has lighter-colored lesions with less infiltration and weaker inflammatory response.  2. Differences in disease progression:  The course of secondary psoriasis is generally shorter and milder than that of psoriasis vulgaris. Some types of secondary psoriasis (such as drug-induced psoriasis) may present with systemic symptoms such as fever and fatigue, whereas psoriasis vulgaris mainly presents with localized chronic lesions.  IV. Lichen planus  1. Characteristics of skin lesions:  Lichen planus lesions are mostly purple-red polygonal papules with shiny surfaces and sometimes form linear patterns. These lesions are often found on flexural surfaces and the oral mucosa and appear as white streaks. Psoriasis vulgaris lesions, on the other hand, are red plaques with silvery-white scales, lacking the purple color and Wickham striae typical of lichen planus.  2. Histopathological differences:  Lichen planus shows degeneration of basal cells and band-like infiltration of lymphocytes in the upper dermis, with colloid body changes in basal cells. Psoriasis vulgaris mainly shows hyperproliferation of epidermal keratinocytes, elongation of rete ridges, parakeratosis, and capillary dilation in dermal papillae with neutrophil infiltration.  ○ Model 4  1. Seborrheic dermatitis  The lesion color is bright red, with few and thin scales, mostly greasy, with unclear borders, and without the thin film phenomenon or punctate bleeding. Commonly occurs on the scalp, eyebrows, behind the ears, armpits, inguinal folds, genital area, and anus.  2. Pityriasis rosea  Commonly occurs on the trunk and proximal limbs, mostly with oval lesions whose long axis is consistent with the direction of skin texture. There is a herald patch or mother patch, without the thin film phenomenon or punctate bleeding.  3. Secondary psoriasis  Lesions are relatively small, mostly light red, with few scales, without the thin film phenomenon or punctate bleeding. The course of the disease is chronic, with no obvious subjective symptoms.  4. Discoid lupus erythematosus  Lesions mainly distribute on the face and limbs, presenting as light red or light purplish-red plaques with clear borders and slightly raised edges. There are adherent scales, and keratotic plugs can be seen after scale removal. Hair follicle atrophy may occur, and the disease progresses chronically, possibly involving internal organ damage. Antinuclear antibodies can be detected in blood tests. Histopathological examination helps with diagnosis.  ○ Model 5  The differential diagnosis of psoriasis vulgaris includes:  1. Seborrheic dermatitis: Should be differentiated from psoriasis vulgaris on the scalp. Seborrheic dermatitis often occurs in areas rich in sebaceous glands, such as the scalp, facial T-zone, chest, and back. The lesions begin as erythematous papules, gradually forming ill-defined red patches covered with fine yellow greasy scales. Hair may become sparse, deformed, or fall out, but alopecia is rare.*  2. Chronic eczema: Should be differentiated from psoriasis vulgaris lesions occurring on the lower legs, anterior tibia, and sacral area. Eczema is often accompanied by intense itching, and the lesions show infiltration, thickening, and lichenification, often with a history of exudation or chronic recurrence.*  3. Neurodermatitis: Should be differentiated from localized plaque-type psoriasis vulgaris. Neurogenic and psychological factors are the main causes. Lesions often occur on the neck, elbow joints, and lumbar region, characterized by pigmentation, lichenification, and thickened plaques covered with a small amount of powder-like scales, differing from the multilayered thick scales of psoriasis vulgaris.*  4. Pityriasis rosea: Should be differentiated from guttate psoriasis. Pityriasis rosea begins with a single pink herald patch or mother patch, followed by multiple oval pink-red patches distributed along the trunk and proximal limbs. Within 1–2 weeks, lesions increase, often showing an oval shape parallel to skin texture, sometimes accompanied by mild itching. The disease is self-limiting, generally subsiding within weeks. Guttate psoriasis lesions have thicker scales and smaller papules but do not follow the same direction as skin texture.*  5. Palmoplantar keratoderma: Should be differentiated from guttate psoriasis. This condition is unrelated to a family history of psoriasis and shows characteristic yellow-red keratinized plaques on the palms and soles, sometimes resembling cracked porcelain. Dermoscopic examination can further assist in differentiation.*  6. Tinea capitis: Should be differentiated from scalp psoriasis. The lesions are covered with grayish-white scales, accompanied by hair loss and broken hair, and fungal spores can be found microscopically, most often seen in children.*  In addition, psoriasis vulgaris should also be differentiated from other psoriasis subtypes, as well as psoriatic arthritis, erythrodermic psoriasis, and pustular psoriasis.*  Note: the asterisk * denotes a source that can be accessed online for further information. |
| Which of the following five large language model responses to the question about the topical treatment of psoriasis vulgaris do you prefer? | ○ Model 1  Psoriasis vulgaris topical treatment mainly includes several types of medications. These help control local inflammation, reduce symptoms, and promote lesion healing. The choice of topical medication depends on the type and severity of the lesion. Common topical medications include:  Topical corticosteroids (glucocorticoids)  Corticosteroids are among the most widely used topical medications for psoriasis vulgaris. They have anti-inflammatory, antipruritic, and vasoconstrictive effects. Based on potency, they can be divided into several categories:  • Strong: such as clobetasol butyrate, fluocinolone.  • Medium: such as triamcinolone acetonide, betamethasone valerate.  • Mild: such as fluocinolone, hydrocortisone, clobetasol propionate.  Usage precautions: Corticosteroids are highly effective for short-term use, but long-term or large-area application can lead to side effects such as skin atrophy, telangiectasia, and systemic absorption. They should be used under medical supervision and discontinued gradually to prevent rebound or worsening.  Vitamin D3 analogs  Vitamin D3 analogs regulate epidermal cell proliferation and differentiation, thereby improving psoriasis lesions. Commonly used agents include:  • Calcipotriol: One of the most common Vitamin D3 analogs used for psoriasis, promoting normalization of keratinocyte proliferation and differentiation to improve psoriatic plaques.  • Tacalcitol: Another Vitamin D3 analog, often used for scalp psoriasis and mild plaques on the trunk.  Usage precautions: Vitamin D3 analogs are generally safe for long-term use, but excessive application can lead to hypercalcemia and should be avoided.  Tar preparations  Tar preparations have anti-inflammatory and antipruritic effects and promote epidermal cell keratinization and desquamation. Common types include:  • Coal tar and wood tar: Common tar preparations used in psoriasis treatment that can reduce inflammation and scaling.  Usage precautions: Tar preparations may cause odor and skin pigmentation; therefore, they are suitable for thick, chronic plaques but not for facial or intertriginous areas.  Calcineurin inhibitors (non-steroidal immunomodulators)  Calcineurin inhibitors, such as tacrolimus and pimecrolimus, are non-steroidal immunosuppressants that inhibit immune inflammatory responses. They are suitable for facial or intertriginous psoriasis, where corticosteroids are less desirable.  • Tacrolimus: Often used for psoriasis of the face and intertriginous regions.  • Pimecrolimus: Suitable for mild to moderate psoriasis.  Usage precautions: Although topical calcineurin inhibitors are generally safe, they can occasionally cause transient burning or irritation.  Keratolytic agents  Keratolytic agents help remove thick scales and promote better absorption of other topical medications.  • Salicylic acid: Commonly used to remove scales and soften the stratum corneum.  Usage precautions: Salicylic acid should not be used on large areas or under occlusion to prevent absorption toxicity, especially in children or severe psoriasis.  Combination therapy  In clinical practice, topical medications are often used in combination—for example, combining corticosteroids with Vitamin D3 analogs—to enhance efficacy and reduce side effects.  Topical immunomodulators  Recently, topical immunomodulators (such as tazarotene and dithranol) have also been used in psoriasis treatment. These agents regulate epidermal proliferation and differentiation and are suitable for mild to moderate psoriasis, though irritation may occur.  Conclusion  Topical treatment is one of the main approaches for psoriasis vulgaris. Commonly used agents include corticosteroids, Vitamin D3 analogs, tar preparations, keratolytic agents, calcineurin inhibitors, and combination therapies. Proper selection and rotation of topical medications can effectively control lesions, relieve symptoms, and improve patients’ quality of life. All topical treatments should be administered under the guidance of a dermatologist.  ○ Model 2  There are many types of topical medications for psoriasis vulgaris. The following are some commonly used topical agents:  Topical corticosteroids  Mild corticosteroids: such as hydrocortisone, desonide, etc*.  Moderate corticosteroids: such as triamcinolone acetonide, betamethasone valerate, etc*.  Strong corticosteroids: such as clobetasol propionate, mometasone furoate, etc*.  Super potent corticosteroids: such as halobetasol propionate, diflorasone diacetate, etc*.  Vitamin D3 analogs  Calcipotriol: suitable for scalp, trunk, and limb areas*.  Tacalcitol: suitable for trunk and stable plaque psoriasis*.  Retinoids  Tazarotene: such as tazarotene gel, suitable for stable plaque psoriasis on the trunk and limbs*.  Retinoic acid: such as 0.025% or 0.1% retinoic acid cream (or gel) *.  Calcineurin inhibitors  Tacrolimus: suitable for facial and intertriginous areas*.  Pimecrolimus*.  Moisturizers  Such as petroleum jelly, silicone oil lotion, vitamin E cream, and urea cream, which help protect the skin and relieve dryness and scaling*.  Keratoplastic and keratolytic agents  Keratoplastic agents: 2%–5% coal tar or ichthammol, 3%–5% sulfur, etc*.  Keratolytic agents: 5%–10% salicylic acid, 10% sulfur, 20% urea, etc*.  Other topical medications  Non-hormonal vitamin E lotion: a non-steroidal topical agent suitable for mild to moderate stable psoriasis in adults*.  Compound preparations: such as combinations of tazarotene with mometasone furoate or calcipotriol with betamethasone dipropionate*.  The choice of topical medication should be based on the patient’s condition, the characteristics of the lesions, and individual needs. When necessary, combination therapy with different mechanisms of action can improve efficacy*.  Note: the asterisk * denotes a source that can be accessed online for further information.  ○ Model 3  The main types of topical medications for psoriasis vulgaris are as follows:  Moisturizers  1. Mechanism of action  Moisturizers can enhance the skin’s barrier function and repair its protective layer. They can reduce scaling, relieve dryness and itching, and improve skin elasticity. By forming a protective film on the skin surface, they prevent water loss and reduce irritation from external stimuli.  2. Common medications  Petrolatum is a common moisturizer. Its chemical properties allow it to form a protective film on the skin surface, preventing water evaporation. Glycerin is another common choice. It has strong hygroscopicity and can absorb water from the air to keep the skin hydrated. Urea cream has high water-retaining capacity, enhances skin hydration, and helps dissolve keratin, promoting exfoliation.  Keratoplastic agents  1. Mechanism of action  Keratoplastic agents promote normalization of keratinization and inhibit excessive proliferation of epidermal cells. These drugs mainly regulate the mitosis and differentiation of epidermal cells, promoting their normalization and improving psoriatic symptoms.  2. Common medications  Tar preparations are traditional keratoplastic agents. Examples include coal tar, which has anti-inflammatory and antipruritic effects. Coal tar can inhibit epidermal DNA synthesis, reduce cell proliferation, and decrease inflammation. Ichthammol has similar effects but is less irritating and suitable for soft lesions such as scalp psoriasis. Salicylic acid is also a common keratoplastic agent. It softens keratin, promotes exfoliation, and is often used for thickened lesions.  Corticosteroids  1. Mechanism of action  Corticosteroids have strong anti-inflammatory and immunosuppressive effects. In psoriasis vulgaris, they reduce inflammation, inhibit immune cell activity, and decrease cytokine release, thereby reducing erythema, scaling, and itching.  2. Common medications  Corticosteroids are classified into weak, moderate, strong, and very strong based on potency. Weak corticosteroids are suitable for facial and intertriginous areas. Moderate corticosteroids like triamcinolone acetonide are widely used and well-tolerated for various lesion sites. Strong and very strong corticosteroids such as clobetasol propionate have potent anti-inflammatory effects but should be used cautiously due to side effects such as skin atrophy and telangiectasia. Generally, for severe lesions, these agents are effective but should not be used long term.  Vitamin D3 analogs  1. Mechanism of action  Vitamin D3 analogs regulate keratinocyte proliferation and differentiation and inhibit inflammatory responses. They bind to vitamin D receptors in cells, promoting normalization of epidermal cell differentiation and reducing hyperproliferation.  2. Common medications  Calcipotriol is the most common Vitamin D3 analog. It is effective for psoriasis vulgaris, especially plaque-type psoriasis, by reducing scaling and redness and improving texture. Tacalcitol is another Vitamin D3 analog with similar effects, suitable for scalp, trunk, and limb psoriasis.  Calcineurin inhibitors  1. Mechanism of action  Calcineurin inhibitors inhibit the activation and cytokine production of T cells, thus exerting anti-inflammatory effects. They block the signaling pathways involved in immune activation, reducing immune-mediated skin damage.  2. Common medications  Tacrolimus and pimecrolimus are typical topical calcineurin inhibitors. Tacrolimus ointment at concentrations of 0.03% and 0.1%, and pimecrolimus 0.03%, are mainly used for facial and intertriginous psoriasis. These agents are effective for delicate skin areas and have a lower risk of steroid-related side effects compared with corticosteroids.  ○ Model 4  1. Tar preparations  Common tar preparations include coal tar, pine tar, ichthammol, and black soybean tar oil, with concentrations generally around 5%. These agents act slowly but have low irritation.  2. Dithranol  Also known as anthralin, this is an important medication for the treatment of psoriasis vulgaris. Common concentrations are 0.1%–1% ointment and 0.5%–2% paste. Treatment usually begins at a low concentration and increases gradually. The medication is applied for 2–4 hours, after which the affected area is cleansed with vegetable oil or soap and water, or coated with vegetable oil to make the medication easier to remove. This method is referred to as short-contact or half-contact therapy.  3. Corticosteroids  These agents have anti-inflammatory, antipruritic, and immunosuppressive effects. They are effective for psoriasis vulgaris but tend to cause relapse after discontinuation. Therefore, they are not used as monotherapy but as part of combination therapy with other agents when the response is poor. Long-term use may cause skin atrophy, telangiectasia, and pigmentation, as well as secondary infections, so caution is required.  4. Retinoic acid  Common forms include 0.05%–0.1% retinoic acid cream or gel and 0.05%–0.1% tazarotene gel. These agents regulate epidermal cell proliferation and differentiation and have mild anti-inflammatory effects. They can be used as monotherapy for mild psoriasis vulgaris or as part of combination therapy for moderate to severe psoriasis vulgaris. These agents may cause some irritation, and treatment should start with low concentrations. Caution is advised when applying to the face or skin folds, and during pregnancy.  5. Calcineurin inhibitors  These agents, such as tacrolimus and pimecrolimus, have strong anti-inflammatory and immunomodulatory effects. They can be used as monotherapy for mild psoriasis vulgaris or as part of combination therapy for moderate to severe cases. These agents may cause mild irritation, and treatment should start with low concentrations. Caution is advised when applying to the face or skin folds.  6. Vitamin D3 analogs  Calcipotriol and tacalcitol are currently approved in China for the treatment of psoriasis vulgaris. These agents inhibit keratinocyte proliferation and promote differentiation. They can be used as monotherapy for mild psoriasis vulgaris or as part of combination therapy for moderate to severe cases. These agents may cause mild irritation, and treatment should start with low concentrations. Caution is advised when applying to the face or skin folds.  ○ Model 5  Topical medications for psoriasis vulgaris include:  1. Corticosteroids: Suitable for various types of psoriasis, with definite efficacy, but not recommended for long-term use to avoid dependence or adverse reactions such as skin atrophy. *  2. Vitamin D3 analogs: Such as calcipotriol and tacalcitol, which regulate epidermal immunity and inhibit excessive keratinocyte proliferation. They are suitable for plaque psoriasis and guttate psoriasis. *  3. Tazarotene: Inhibits keratinocyte DNA synthesis and induces cell differentiation. It also has anti-inflammatory and cytokine-inhibitory effects and is suitable for chronic plaque psoriasis and guttate psoriasis. *  4. Dithranol: Inhibits keratinization and keratinocyte DNA synthesis and induces cell differentiation. It is suitable for chronic plaque psoriasis and guttate psoriasis. *  5. Fluorouracil: Has anti-metabolic effects and is used for localized psoriatic lesions, but caution should be taken regarding possible skin irritation and pigmentation. *  6. Cyclosporine: Has immunosuppressive effects and is used in the treatment of psoriasis, but caution is needed for possible skin irritation and photosensitivity. *  7. Coal tar: Has keratoplastic and keratolytic effects and is used for chronic plaque psoriasis, but caution is required for possible clothing staining, tar odor, and adverse effects such as contact dermatitis and folliculitis. *  8. Tacrolimus: Has strong anti-inflammatory and immunosuppressive effects and is used for thick plaque psoriasis, but caution is required for possible irritation and photosensitivity. *  In addition, there are many other topical options, such as pimecrolimus and tacalcitol. The choice of topical therapy should be based on the patient’s condition and physician’s recommendation. *  Note: the asterisk * denotes a source that can be accessed online for further information. |
| Which of the following five large language model responses to the question about the case study of psoriasis do you prefer?  Question: A 36-year-old male patient presents with a several-year history of recurrent red patches and scales on the body, which worsen during autumn and winter and improve during spring and summer. Six months ago, the lesions increased again, appearing on the scalp, trunk, and limbs, accompanied by itching and dryness. Based on this medical history and examination findings, what is the most likely diagnosis? | ○ Model 1  Based on the provided medical history and examination results, the most likely diagnosis is psoriasis vulgaris (Psoriasis vulgaris). The detailed diagnostic basis is as follows:  1. Medical history  Onset pattern: The patient has a long history of recurrent skin lesions with seasonal fluctuations—aggravated in autumn and winter, alleviated in spring and summer.  Triggering factors: The disease relapsed after a cold, consistent with the pattern of psoriasis exacerbation following infections or external stimuli.  Family history: No family history of psoriasis or similar conditions, but this does not exclude the diagnosis.  2. Physical examination  Skin lesions: Scattered erythematous plaques covered with silvery-white scales, showing clear boundaries and typical psoriatic morphology.  Classic signs:  Auspitz sign: Punctate bleeding appears after scales are removed.  Film phenomenon: A translucent membrane is observed after removing scales.  Punctate bleeding: Small bleeding points appear after removing the film.  Distribution: Symmetrical lesions mainly on the scalp, trunk, and limbs, especially the extensor surfaces of elbows and knees.  Nail changes: Pitting and thickening observed, consistent with psoriatic nail changes.  3. Typical dermatological findings Erythema, scaling, and characteristic Auspitz sign are typical features of psoriasis vulgaris.  4. Exclusion of other diseases  Seborrheic dermatitis: Limited to the scalp and oily areas with greasy scales, not as thick as psoriasis.  Pityriasis rosea: Self-limiting, short course, and specific distribution, unlike psoriasis.  Eczema: Characterized by vesicles and exudation in the acute stage, unlike the chronic dry plaques seen in psoriasis.  Dermatophytosis: Fungal examination negative, excluding fungal infection.  5. Conclusion  Combining the medical history, clinical features, and examination findings, the most likely diagnosis is psoriasis vulgaris (Psoriasis vulgaris). This is a chronic, recurrent inflammatory skin disease characterized by erythematous plaques covered with silvery-white scales, often worsening in cold and dry seasons and improving in warm and humid conditions.  ○ Model 2  The most likely diagnosis is psoriasis vulgaris. The diagnostic basis is as follows:  The patient presents with generalized red plaques covered by thick layers of scales, showing candle grease phenomenon, film phenomenon, and punctate bleeding, which are characteristic features of psoriasis vulgaris*.  The patient has a 5-year disease history with annual recurrences and prior use of topical and herbal treatments, consistent with the chronic relapsing nature of psoriasis*.  Thickened plaques with scales were observed on the frontal hairline and scalp, showing typical scalp involvement of psoriasis*.  The absence of facial and mucosal involvement is consistent with the usual distribution pattern of psoriasis vulgaris*.  Note: the asterisk * denotes a source that can be accessed online for further information.  ○ Model 3  The most likely diagnosis: Psoriasis vulgaris.  Diagnostic basis:  1. Medical history  The patient has a history of recurrent attacks. The first onset occurred five years ago after overwork during the winter, and recurrences followed annually. This is consistent with the chronic and relapsing characteristics of psoriasis vulgaris.  2. Clinical manifestations  Characteristics of skin lesions:  Red plaques scattered on the trunk and limbs, covered with thick scales, showing the typical candle grease phenomenon (silvery-white scales, thick and shiny), film phenomenon (a translucent film appearing after removing scales), and punctate bleeding (Auspitz sign). These are classic features of psoriasis vulgaris.  Thickened plaques with scales found on the frontal hairline and scalp, with patchy distribution. This represents typical scalp involvement seen in psoriasis vulgaris.  Distribution pattern:  The absence of facial and mucosal involvement aligns with the usual distribution pattern of psoriasis vulgaris, which predominantly affects the scalp, trunk, and limbs, especially the extensor surfaces.  ○ Model 4  The most likely diagnosis is psoriasis.  Diagnostic basis:  1. Medical history: The patient is 36 years old, with a disease course of 5 years. The lesions recur repeatedly, consistent with the chronic and relapsing characteristics of psoriasis.  2. Clinical manifestations: Red plaques distributed throughout the body, covered with thick scales, showing candle grease phenomenon, film phenomenon, and punctate bleeding positivity, which are typical clinical features of psoriasis.  3. Special site involvement: Thickened plaques with scales are visible on the frontal hairline and scalp, with patchy distribution, consistent with the clinical characteristics of psoriasis.  ○ Model 5  Based on the provided information, the most likely diagnosis is psoriasis vulgaris.*  Diagnostic basis:  1. Medical history: Five years ago, after overwork during the winter, red rashes appeared all over the body. The patient was treated with traditional Chinese medicine decoctions and topical medications. The lesions gradually subsided but recurred annually. There is no family or genetic history of similar diseases, and no history of drug allergy or infectious disease.  2. Dermatological examination: Red plaques distributed on the trunk and limbs, covered with thick scales, showing candle grease phenomenon, film phenomenon, and punctate bleeding. Thickened plaques with scales were scattered on the frontal hairline and scalp, with patchy distribution. The lesions were patchy, and facial and mucosal areas were not involved.  The medical history and dermatological findings are consistent with the characteristics of psoriasis vulgaris; therefore, the diagnosis is psoriasis vulgaris.*  Note: the asterisk * denotes a source that can be accessed online for further information. |
| Part 3: Please rate the importance of the following five dimensions in the medical large language model response. (Only one dimension can be selected as “very important.”)  ○ Accuracy: whether the content is correct and free from factual errors.  ○ Comprehensiveness: whether the response fully covers all relevant aspects of the question.  ○ Conciseness: whether the response is succinct and avoids unnecessary redundancy.  ○ Logicality: whether the content is logically organized, coherent, and readable.  ○ Traceability: whether the response provides references or sources (e.g., websites, literature, or clinical guidelines | |
| (1) Accuracy | ○ Very unimportant  ○ Unimportant  ○ Slightly important  ○ Moderately important  ○ Important  ○ Very important |
| (2) Comprehensiveness | ○ Very unimportant  ○ Unimportant  ○ Slightly important  ○ Moderately important  ○ Important  ○ Very important |
| (3) Conciseness | ○ Very unimportant  ○ Unimportant  ○ Slightly important  ○ Moderately important  ○ Important  ○ Very important |
| (4) Logicality | ○ Very unimportant  ○ Unimportant  ○ Slightly important  ○ Moderately important  ○ Important  ○ Very important |
| (5) Tracebility | ○ Very unimportant  ○ Unimportant  ○ Slightly important  ○ Moderately important  ○ Important  ○ Very important |

Note: The original questionnaire was administered in Chinese. The English version provided here is a translation of the original Chinese questionnaire, and no changes have been made to its meaning.
